# Supplementary material for: Behavioral evolution contributes to hindbrain diversification among Lake Malawi cichlid fish
Source: Sci Rep. 2019 Dec 27;9:19994. doi: 10.1038/s41598-019-55894-1 (PMC6934501; doi:10.1038/s41598-019-55894-1)
Supplement: Supplementary file 1 — Supplementary materials and methods [file 41598_2019_55894_MOESM1_ESM.docx]

Behavioral evolution contributes to hindbrain diversification among

Lake Malawi cichlid fish

**Authors:** Ryan A. York^1,2*^, Allie Byrne^1^, Kawther Abdilleh^3^, Chinar Patil^3^, Todd Streelman^3^, Thomas E. Finger^4,5^, Russell D. Fernald^1,6^

**Affiliations:**

^1^Department of Biology, Stanford University, Stanford, California 94305, USA

^2^Department of Neurobiology, Stanford University, Stanford, California 94305, USA

^3^School of Biological Sciences and Institute of Bioengineering and Bioscience, Georgia Institute of Technology, Atlanta, Georgia 30332, USA

^4^Department of Cell and Developmental Biology, University of Colorado Denver, Aurora, CO 80045

^5^Rocky Mountain Taste and Smell Center, University of Colorado Denver, Aurora, CO 80045

^6^Neuroscience Institute, Stanford University, Stanford, California 94305, USA

^*^Correspondence: [ryanyork@stanford.edu](mailto:ryanyork@stanford.edu)

**Supplemental experimental procedures**

**1. Animals**

Male and female *Copadichromis virginalis*, *Mchenga conophoros*, and *Tramitichromis brevis* individuals were housed in 50-gallon rectangular tanks that with a uniform 40 lb layer (~ 4 cm in depth) of Malawi sand (CaribSea Inc.; Sahara Sand, 00254). All species were grown and maintained in laboratory conditions mimicking natural pH (8.2), conductivity (230uS) and temperature (26°C) in a 12:12 hour light:dark cycle. Fish were housed and maintained under established Stanford University IACUC protocols.

**2. Phylogenetic comparative methods**

***Brain volume calculation***

All volumetric analyses were performed using measurements of height, length, and width for six brain regions (telencephalon, hypothalamus, cerebellum, optic tectum, olfactory bulb, and hindbrain excluding cerebellum) from a phenotypic data set comprising brain measurements for 189 cichlid species from East Africa and Madagascar [19]. Region volumes (V) were calculated using the following ellipsoid model:

V = 1/6𝜋abc

where a represents length, b represents height, and c represents width. All normalized region volumes measures were produced by dividing raw volumetric measures by the reported standard length (in millimeters) of the species sample.

***Phylogenies***

We obtained genome-wide SNP calls for Malawi cichlids [21] (BioProject: PRJNA380805).

To produce the Malawi phylogeny SNPhylo [32] was run for 1,000 bootstrap rounds, default parameters were used otherwise. The chronos function in the R package ape was used to produce ultrametric and rooted phylogenies for analyses in which these were required [33]. The species *Astatotilapia calliptera* was used as outgroup for rooted trees. The calibration points of 1.6 MYA (*Astatotilapia calliptera*/mbuna+sand) and 0.57-1 MYA (mbuna/sand) were used to create all Malawi ultrametric trees [34-35]. To produce the trees chronos was run with a relaxed model and a lambda value of 1.

Lake Tanganyika analyses used the best ultrametric maximum likelihood phylogeny from [36]. We added the following calibration points to the Tanganyika tree per Day et al. 2008: Bathybatini 8.7-11.1 MYA, C-lineage 8.0-10.6 MYA, L-lineage 7.9-10.8, Limnochromini 3.0-6.1 MYA, Cyprichromini 3.3-6.5 MYA, and Ectodini 5.4-7.5 MYA. Bower designations for Lake Tanganyika species were taken from [37]. Bower designations for Lake Malawi species were taken from [17] and [38].

***Analyses***

Phylogenetic ANOVAs were performed using the aov.phylo command in the R package geiger [39]. All Malawi cichlid phylogenetic ANOVAs used normalized trait measures and the respective phylogenetic trees produced above. Measures of phylogenetic signal (Blomberg’s *K*; Pagel’s λ) were computed using the phyloSignal command in the R package phylosignal [40]. Trait diversification rates were measured using Bayesian Analysis of Macroevolutionary Mixtures (BAMM) v.2 [23] and analyzed and visualized in R using the package BAMMtools. For both the Tanganyika and Malawi analyses hindbrain diversification rates were computed using the ‘trait’ model and run for 100 million generations. These runs resulted in effective sample sizes of 6538.17 (Malawi) and 539.54 (Tanganyika), suggesting that the MCMC simulation converged. Rate differences between clades (as in figs. 1g and 6f) were compared by extracting trait diversification rates (beta) for each tip of the respective trees and performing a Kruskal-Wallis test.

***Window based tests***

To perform the window based phylogenetic tests the genome-wide SNP calls for both Malawi sand and rock-dwelling species were filtered to just biallelic SNPs using VCFtools [41]and then phased with Beagle [42] allowing for imputation and using a window size of 10000 and an overlap of 1000. The resulting variants were converted to genotypes with the python function parseVCF.py from which maximum likelihood phylogenies were produced for 10000 variant windows using phyml via the python function phyml_sliding_windows.py (both functions are available at github.com/simonhmartin/genomics_general). The resulting 1,029 phylogenetic trees were visualized in R using custom code. As above phylogenetic ANOVAs were performed on each window using aov.phylo and measures of phylogenetic signal were calculated with phyloSignal.

**3. Immunohistochemistry and DiI experiments**

For DiI tracing, the crania including the brain were rinsed in buffer and dissected to expose the dorsal brainstem and cranial nerve roots. Crystals of DiI then were placed either onto the vagus nerve root(s) or onto the surface of the vagal lobe. Low melting point agar was applied to hold the crystals in place and to avoid incidental spread to other neural structures. The specimens then were returned to fixative and remained at room temperature for 1 – 6 months to allow diffusion of the diI along the length of the labeled axons.

After this diffusion period, the brains were dissected free of the skull, washed in buffer and sectioned at 75-100 um on a vibratome. Sections were immediately mounted, coverslipped with Fluoromount (Sigma-Aldrich, St. Louis MO, F4680) and photographed.

**4. Functional anatomical assays**

***Animals used***

Behaviourally-induced neural activity of adult males from the species *M. conophoros* and *C. virginalis* was assayed with both *in situ* hybridization and immunohistochemical labeling after one of three behavioural paradigms: court/build (*in situ*: n = 3; immuno: n = 4), starvation (*in situ*: n = 3; immuno: n = 3), or control (*in situ*: n = 3; immuno: n = 3).

***Behavioural paradigms:***

***Court/build paradigm***

Adult *M. conophoros* and *C. virginalis* males were housed with one other male and 5-8 females in a 40-gallon tank. A GoPro camera (GoPro; Hero4 Silver, CHDHY-401) housed in a waterproof compartment (GoPro; Clear Standard Housing, AHSRH-401) was placed in front of the tank and fish were monitored from another room. Observation began at upon lights turning on in the morning until males began building. All observations were performed at the same time of day (as with the starvation and control paradigms) and all fish demonstrated building behaviour within two hours of lights on. Building behaviour was classified as moving sand from many locations around the tank on top of the bower in a characteristic circle pattern indicative of castle-building (*M. conophoros*) or moving mouthfuls from a depression in the sand to the edges as demonstrated during pit-digging (*C. virginalis*). Fish were considered to be constructing bowers if they built/dug more than 10 times in 10 minutes and built for more than 15 minutes. Focal males were sacrificed at either 30 minutes after initiation of behaviour (for *in situ* hybridization) or 60 minutes after initiation (for immunohistochemistry) in accordance with the differential rates of mRNA and protein accumulation.

***Starvation paradigm***

Adult *M. conophoros* and *C. virginalis* males were housed individually in 40-gallon tanks divided in half by a black, opaque plexiglass divider. Males were left in one compartment of the tank for 3 days, without food. A GoPro camera was placed in front of the tank to allow monitoring of the fish remotely. On the fourth day, airlines and water filtration were turned off to prevent flakes from being disturbed. Spirulina flakes were buried under a thin layer of sand, along the bottom half of the tank that the fish did not occupy. The barrier was then raised and fish were able to swim freely within the entire tank. Fish that freely fed over the course of at least 15 minutes were then sacrificed at either 30 minutes (for *in situ* hybridization) or 60 minutes (for immunohistochemistry) after initiation of behaviour.

***Control paradigm***

Two adult males of the species *M. conophoros* or *C. virginalis* were housed overnight in an 8-gallon tank with a clear, perforated plexiglass divider separating them from each other. Fish were monitored via a live-streaming GoPro in order to monitor their activity levels without disruption by the observation. Males were then sacrificed the following morning at the same hour as the bower building and starvation paradigms.

***Histological preparations:***

**In situ *hybridization***

We used RT-PCR to amplify a portion of coding sequence from *cFos* (NM_001286320), and subcloned products into pCR-TOPO4 (Life Technologies). *cFos* forward, 5’-AAT TGG ATC CAA GCC CAG ATC TTC AGT GG-3’; *cFos* reverse, 5’-AAT TGA ATT CAT AGC CCT GTG ATC GGC AC-3’. Antisense RNA probe was transcribed using T7 (*cFos*; Promega) RNA polymerase. Brains were collected 30 minutes after the start of a behaviour and placed in 4% PFA overnight at 4C. Brains were then transferred to 30% sucrose overnight at 4C and embedded and stored at -80C. *In situ hybridization* was performed as described previously (Ma et al 2015) except that slides were incubated in BCIP/NBT overnight.

***Immunohistochemistry***

Brains were dissected 90 minutes after the start of a behaviour and placed in 4% PFA for 4 hours at room temperature. Brains were then transferred to 30% sucrose overnight at 4C. Brains were embedded in Neg50 and stored at -80C. Brains were sectioned at 30um thickness. Slides were immersed in boiling citric acid (10 mM, pH 6) for 5 minutes twice. Slides were washed in 1x PBS, then incubated in blocking solution (1x PBS, 5% Donkey Serum, 0.3% Triton). Slides were then incubated in PBST (1x PBS, 0.5% Donkey Serum, and 0.3% Triton) with primary antibodies overnight at room temperature. Rabbit anti Phospho-S6 Ser244 Ser247 (ThermoFIsher Scientific 44-923G, RRID [AB_2533798](http://antibodyregistry.org/AB_2533798), lot SE251246, at 1:300) was used. The amino acid sequence of the final 20 amino acids of zebrafish ribosomal S6 surrounding the identified phosphorylation site is 100% identical to the human peptide used for antibody generation.

The next morning, slides were washed in 1x PBS and incubated in PBST and secondary antibodies Donkey anti-goat (ThermoFisher Scientific, Alexafluor 594, A11058, 1:300) and Donkey anti rabbit (ThermoFisher Scientific, Alexafluor 488, A21206, 1:300) for 1 hour. Slides were rinsed in PBS with DAPI and coverslipped with aquapolymount. Images were acquired with a Nikon Eclipse Ni-E and processed using FIJI [43, 44].

***Histological quantification:***

***cFos* expression quantification**

Quantification of *cFos* *in situ* hybridization (ISH) staining was performed in FIJI. First, a region of interest with staining was drawn around the vagal lobe. The image was next inverted and mean pixel intensity and ROI area were calculated. This process was then repeated in a bordering region of the hindbrain on the same. N=Mean intensity values in the bordering region were subtracted from the ROI in order to calculate the pixel intensity over background for each ROI. The resulting pixel intensities were then averaged within each brain. Given the moderate sample size for each group the resulting mRNA expression measures were compared using a bootstrap 1-way ANOVA run for 10,000 rounds in R.

***pS6 quantification***

Quantification of pS6 labeling was performed using FIJI. Images were loaded and converted to 8 bit. In order to remove background signal the images were thresholded using the ‘triangle’ autothresholding method. The images were then converted to binary and watershed separation was applied in order to separate closely oriented objects. A region of interest was then delineated around the dorsal vagal lobe and pS6 positive cells were counted using the Analyze Particles plugin. Cell counts were done unilaterally and were summed up for each sample for each vagal lobe section that showed positive labeling. Counts were dong blinded to experimental condition. The resulting summed pS6 positive cell counts were then compared using a bootstrap 1-way ANOVA run for 10,000 rounds in R.


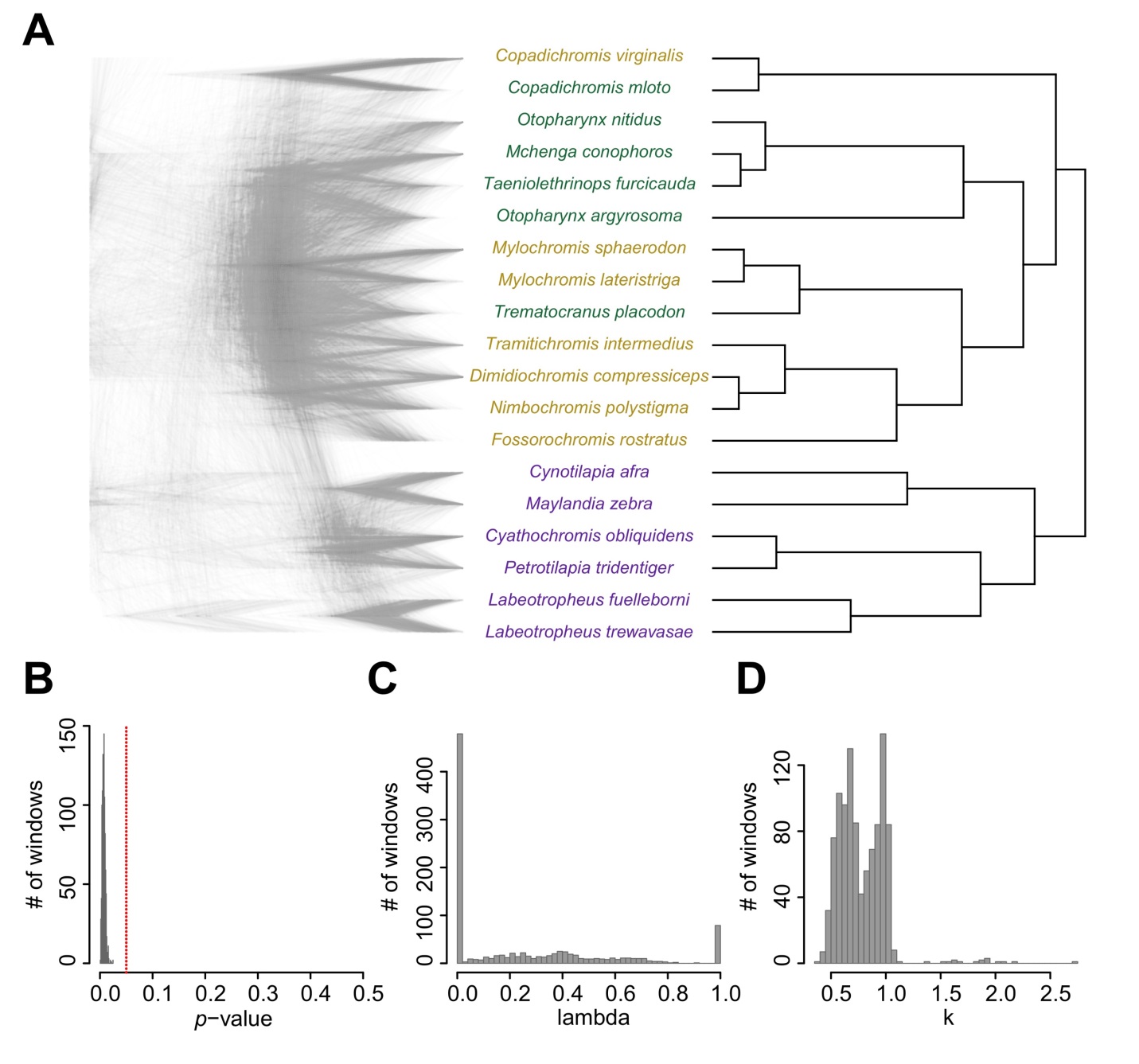


**Fig S1. Window-based phylogenetic tests**

**(A)** The leftward facing multiple-phylogeny plot includes all 1,029 windows sampled from 10,000 variant sets. The rightward facing tree was produced from whole-genome SNP information. Species names colored green are castle-builders, yellow are pit-diggers, and purple are rock-dwelling species. **(B)** Histogram of *p-*values resulting from phylogenetic ANOVAs on all 1,029 windows (red line represents *p*-value = 0.05). **(C-D)** Histograms of Pagel’s λ and Blomberg’s *K* values, respectively, calculated from the 1,029 genomic windows.


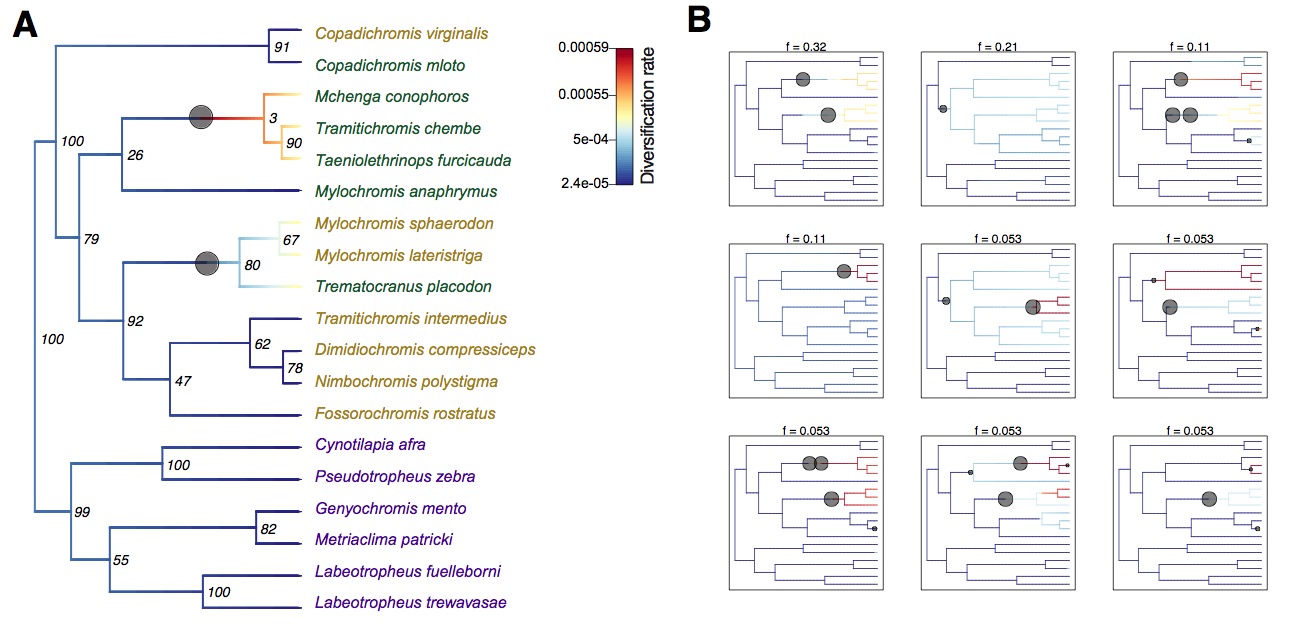


**Fig S2. BAMM analyses**

**(A)** Phylogeny of best supported rate shifts from BAMM Branch colors indicate diversification rate. The two transparent circles represent the locations of the two best supported rate shifts among the sand and rock lineages. **(B)** 95% credible set of rate shifts from BAMM. Branch colors indicate diversification rate. The two transparent circles represent the locations of the two best supported rate shifts among the sand and rock lineages. Posterior probabilities are included above each plot. The first four shift sets cover the majority of posterior probabilities sampled. Three of these four include significant diversification rate increases on at least one of the two castle-builder dominated branches identified by the most credible rate shift set.

**Figure S3. DiI labeling of the vagal lobe**

(**A-F) *Copadichromis virginalis*.** Series of transverse sections through the vagal lobe of *C. virginalis*showing DiI labeling of the vagus nerve root from **(A)** anterior to **(F)** posterior.  Numerals indicate inter-section intervals in microns. The sensory roots of the nerve (NXs) split into superficial and deep branches terminating in respective superficial and deep terminal zones. Labeled nerve fascicles run from the superficial layers into the deeper layer throughout the length of the lobe. The motor nucleus of the glossopharyngeal nerve (NIXm) and  vagus nerve (NXm) run ventrally, taking their origin from the motor neurons (mn) situated along the ventricular surface ventral to the vagal lobe proper. **E’** shows a 2X enlargement of the vagal motor pool at level adjacent to that shown in panel **(E)**. **(G-L) *Mchenga conophoros***. Series of transverse sections through the vagal lobe of *M. conophoros*showing DiI labeling of the vagus nerve root from **(G)** anterior to **(L)** posterior.  Numerals indicate inter-section intervals in microns. The sensory roots of the nerve (NXs) split into superficial and deep branches terminating in respective superficial and deep terminal zones. Labeled nerve fascicles run from the superficial layers into the deeper layer throughout the length of the lobe. The motor nucleus of the vagus nerve (NXm) runs ventrally, taking its origin from the motor neurons (mn) situated along the ventricular surface ventral to the vagal lobe proper. Inset **J’** shows labeled motoneurons in an adjacent section.


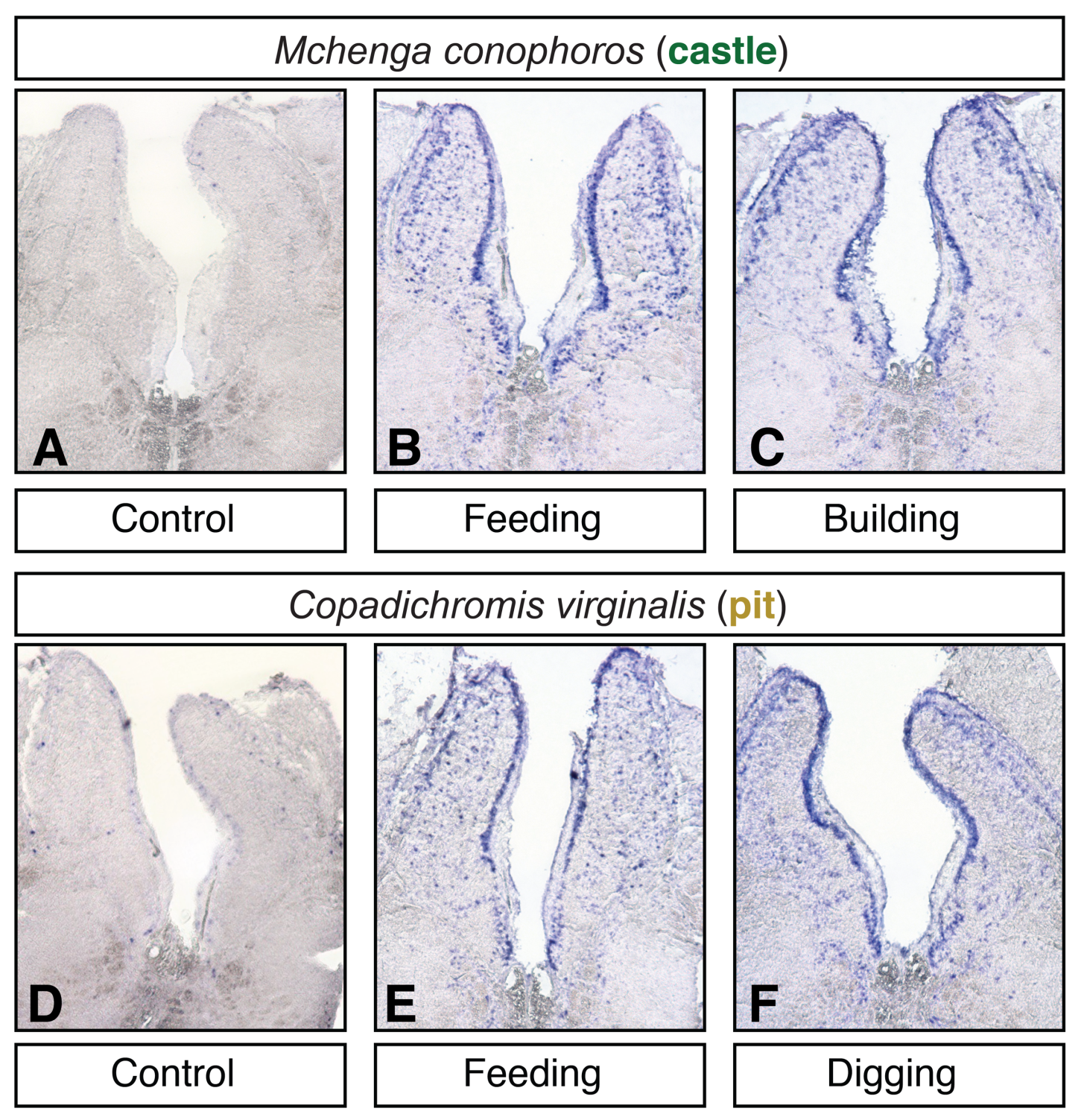
**Fig S4. *cFos* mRNA expression during behaviour in the vagal lobe**

**(A)** In situ hybridization of *cFos* mRNA in the vagal lobe (VL) of *Mchenga conophoros* (MC) male subjected to the control behavioral paradigm. **(B)** *cFos* mRNA expression in the VL of a feeding MC male. **(C)** *cFos* mRNA expression in the VL of a castle building MC male. **(D)** *cFos* mRNA expression in the VL of a control *Copadichromis virginalis* (CV) male. **(E)** *cFos* mRNA expression in the VL of a feeding CV male. **(F)** *cFos* mRNA expression in the VL of a pit digging CV male.


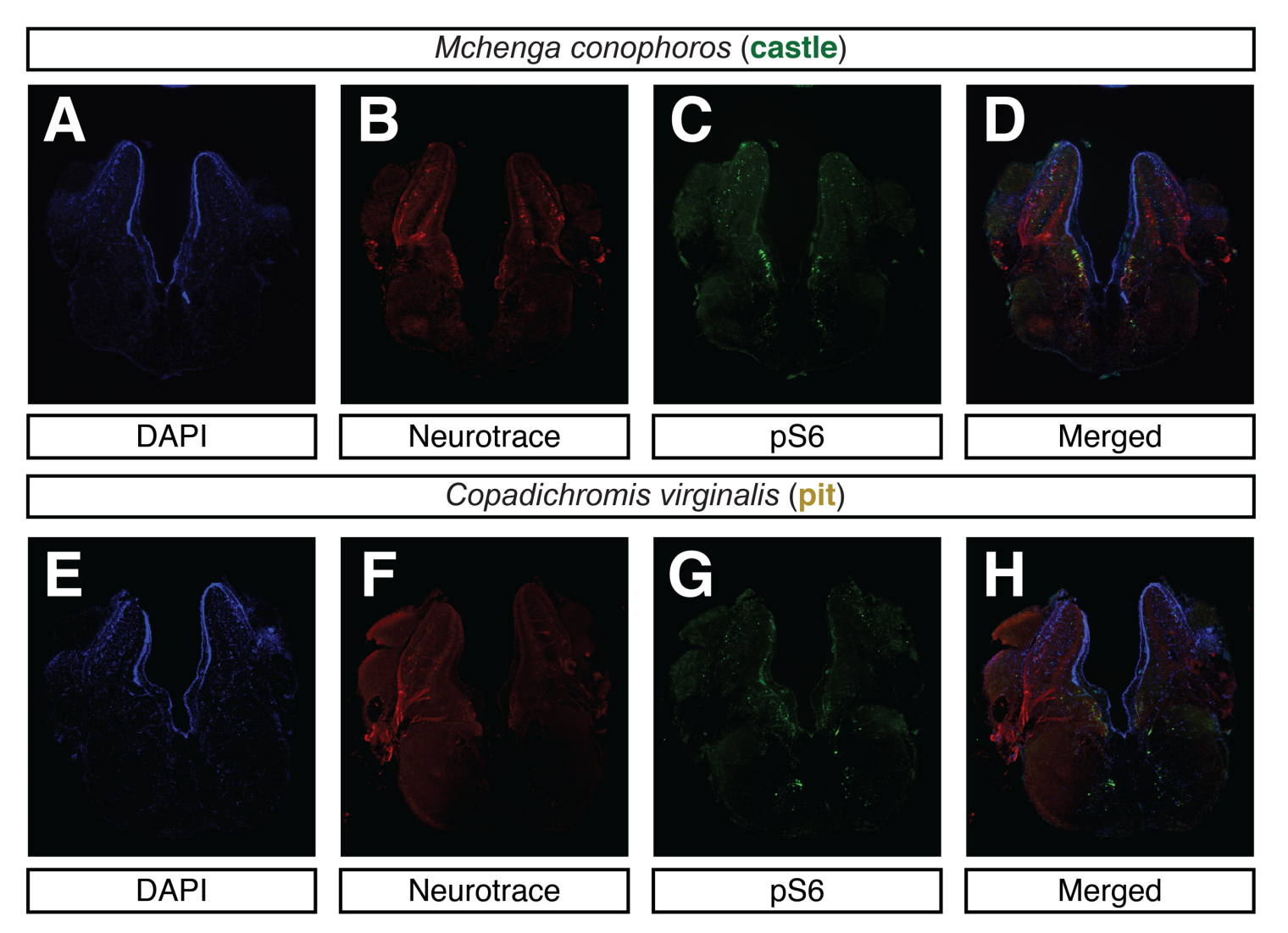


**Fig S5. pS6 abundance associated with bower building in the vagal lobe**

**(A)** DAPI labeling of cell nuclei in the VL of *M. conophoros.* **(B)** Neurotrace labeling of nissl substance in *M. conophoros* VL neurons. **(C)** Fluorescent immunohistochemical labeling of pS6 in *M. conophoros* VL. **(D)** Merge of panels **(A-C)**. **(E)** DAPI labeling of cell nuclei in the VL of *C. virginalis.* **(F)** Neurotrace labeling of nissl substance in *C. virginalis* VL neurons. **(G)** Fluorescent immunohistochemical labeling of pS6 in *C. virginalis* VL. **(H)** Merge of panels **(E-G)**.

**References**

37. Konings, A. *Tanganyika Cichlids in their Natural Habitat: 3rd Edition*. (Hollywoord Import & Export Inc., Florida 2015).

38. Konings, A. *Malawi Cichlids in their Natural Habitat, 4th Edition*. (Cichlid Press 2007).

39. Harmon, L. J., J. T. Weir, C. D. Brock, R. E. Glor, & W. Challenger. GEIGER: investigating evolutionary radiations. *Bioinformatics*. **24**, 129-131 (2008).

40. Keck, F., Rimet, F., Bouchez, A., & Franc, A. phylosignal: an R package to measure, test, and explore the phylogenetic signal. *Ecology and Evolution.* **6**, 2774-2780 (2016).

41. Danecek, P. *et al*. The variant call format and VCFtools. *Bioinformatics*. **27**, 2156-2158 (2011).

42. Browning, S.R., & Browning, B.L. Rapid and accurate haplotype phasing and missing data inference for whole genome association studies by use of localized haplotype clustering. *Am. J. Hum. Genet.* **81**, 1084-1097 (2007).

43. Schindelin, J. Fiji: an open-source platform for biological-image analysis. *Nat. Methods.* **9**, 676-682 (2012).

44. Schneider, C.A., Rasband, W.S., & Eliceriri, K.W. NIH Image to ImageJ: 25 years of image analysis. *Nat. Methods.* **9**, 671-675 (2016).

45. Mckaye, K.R. *et al*. Fishes, as well as birds, build bowers. *J. Aquaricult. Aqua.t Sci.* **9**, 121-129 (2001).

46. Konings, A. *Tanganyika Cichlids in their Natural Habitat: 3rd Edition* (Florida: Hollywoord Import & Export Inc., 2015).

47. Stauffer, J.R., & Konings, A. Review of Copadichromis (Teleostei: Cichlidate) with the description of a new genus and six new species. *Ichthyol. Explor. Freshwaters*. **7**, 9-42 (2006).

48. Kellog, K.A., Markert, J.A., Stauffer, J.R., & Kocher, T.D. Intraspecific brood mixing and reduced polyandry in a maternal mouth-brooding cichlid. *Behavioral Ecology.* **9**, 309-312 (1998).

49. Kidd, M.R., Kidd, C.E., & Kocher, T.D. Axes of differentiation in the bower-building cichlids of Lake Malawi. *Mol. Ecol.* **15**, 459-478 (2006).
